# The “Levine effect” and the father of modern diabetes research

**DOI:** 10.1016/j.jbc.2021.101356

**Published:** 2021-10-28

**Authors:** Kenneth T. Farabaugh

Even just over a century ago, a diagnosis of diabetes was practically a death sentence. There was no treatment but to attempt to extend the survival of those afflicted *via* a so-called ‘starvation diet’; as a result, parents and doctors could do nothing but watch as their children and patients wasted away, succumbing to starvation, ketoacidosis, or to numerous other complications of diabetes such as heart disease, infections, and kidney failure. The discovery of insulin and its use in humans was revolutionary and has saved countless lives ever since that first fateful injection by Banting and Best in 1921. However, the basic science surrounding insulin at that time was rudimentary—it was not at all known what insulin even was, other than some mysterious glandular extract from Langerhans’ so-called ‘islets of cells’ in the pancreas, let alone how it worked.

The first theory of the mechanism of action of insulin, put forth by Vilém Laufberger in 1924, was that insulin acted covalently on enzymes to directly block the process of gluconeogenesis and thereby prevented the body from producing excess glucose to circulate in the blood (1, 2). This theory dominated the field for a number of years, and the established researchers took for granted that insulin and glucose were freely able to diffuse into cells. Around this time, Rachmiel Levine, a young Polish-born orphan, was denied a visa to enter the United States and instead emigrated to Canada (3). Levine later entered McGill University in Montreal, graduating with an MD in 1936, and interned at Michael Reese Hospital in Chicago before becoming the assistant director of their Department of Metabolic and Endocrine Research in 1939. He published several articles there with William Soskin on the relationship between blood-sugar levels and glucose utilization (4, 5), as well as a book entitled “Carbohydrate Metabolism” (6), cementing his research pedigree and his focus on the role of insulin.

At the time, biological research on animals was less regulated, and not for the squeamish. The preferred diabetic animal model of choice was dogs in which the pancreas and kidneys had been removed, which mimicked the pathological conditions of human type I diabetes. Using this model, Levine *et al.* (7) set out to discover the true mechanism of insulin action. By injecting these dogs with galactose, a hexose sugar similar to glucose but which is not readily metabolized in this model, they found that 45% to 47% of the galactose was taken up by cells into body tissues. However, upon coinjection of the same total amount of galactose along with insulin, the plasma levels of galactose were much lower. This result indicated that insulin prompted cells in body tissues to take up more of the galactose.

**Figure 1:**
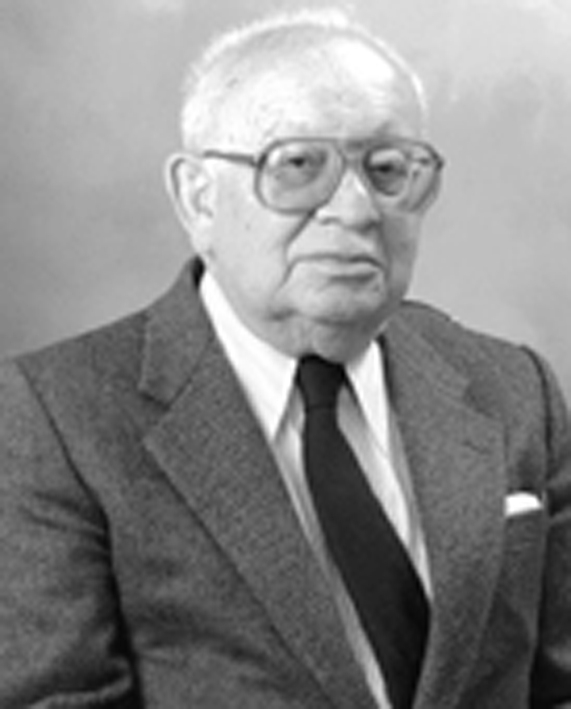
**Rachmiel Levine, the father of modern diabetes research.** Photo from the European Association for the Study of Diabetes.

Based on this finding, Levine *et al.* formulated the theory that made their careers, namely that insulin acted on the cell membrane to promote sugar uptake from the blood. This was eventually termed “the Levine effect,” and the publication of this finding in the JBC in 1949 (7) is generally recognized as the origin of his nickname, “the father of modern diabetes research” (Fig. 1). Levine’s transport theory was soon after confirmed using xylose and arabinose (8, 9, 10, 11), as well as glucose (12, 13), and in striated (14, 15, 16) and cardiac muscle cells (17, 18), fibroblasts (19), and adipose tissue (20), thus generalizing the action of insulin on the uptake of sugars by many cell types. This work was followed by studies showing that insulin itself did not need to be taken up into cells to exert these effects, bucking the established theory of the day that that glucose and insulin could freely cross the cell membrane. It was clear that there was some mechanism or mechanisms that regulated and maintained the uptake and maintenance of hexose levels in cells, although it was not clear yet what this mechanism might be.

While it did not account for many of the secondary actions of insulin (21), Levine’s theory of insulin working on cell membranes was a breakthrough in diabetes research. In subsequent years, many studies were published on the mechanism of insulin action, including the identification of the insulin receptor (22), which confirmed the extracellular role of insulin in mediating glucose uptake. Levine spent the next 45 years at Michael Reese and at the University of Chicago, then as a professor and chairman at New York Medical College, and finally at City of Hope Medical Center in Duarte, California, publishing his research on diabetes and built their Diabetes Program. In 1978, he also encouraged two fellow researchers Arthur Riggs and Keiichi Itakura at City of Hope to genetically engineer *Escherichia coli* to produce human insulin (23); this led to the development of Humulin, the first genetically engineered health-care product approved by the Food and Drug Administration, which is still used today by millions of diabetics (https://www.cityofhope.org/research/levine-symposium/about-rachmiel-levine, accessed October 18, 2021).

Looking back on the 1949 study covering just two pages in the JBC (7), it is truly amazing the wonders that would come from such a short experiment: a foundational discovery challenging the beliefs of the field, a lasting legacy of research integrity, and a namesake effect that cemented Rachmiel Levine as the true father of modern diabetes research.
